# Effect of edaravone on radiation-induced brain necrosis in patients with nasopharyngeal carcinoma after radiotherapy: a randomized controlled trial

**DOI:** 10.1007/s11060-014-1573-4

**Published:** 2014-08-21

**Authors:** Yamei Tang, Xiaoming Rong, Weihan Hu, Guoqian Li, Xiaoxia Yang, Jianhua Yang, Pengfei Xu, Jinjun Luo

**Affiliations:** 1 Department of Neurology, Sun Yat-Sen Memorial Hospital, Sun Yat-Sen University, Number 107, Yan Jiang Xi Road, Guangzhou, 510120 Guangdong China; 2 Key Laboratory of Malignant Tumor Gene Regulation and Target Therapy of Guangdong Higher Education Institutes, Sun Yat-Sen University, Guangzhou, China; 3 Department of Radiation Oncology, Cancer Canter of Sun Yat-sen University, Guangzhou, China; 4 Department of Neurology, Fujian Provincial Quanzhou First Hospital, Quanzhou, Fujian China; 5 Departments of Neurology and Pharmacology, Temple University School of Medicine, Philadelphia, PA USA

**Keywords:** Radiation-induced brain necrosis, Edaravone, Corticosteroid, Nasopharyngeal carcinoma

## Abstract

Excessive generation of free radicals plays a critical role in the pathogenesis of radiation-induced brain injury. This study was designed to evaluate the protective effect of edaravone, a free radical scavenger, on radiation-induced brain necrosis in patients with nasopharyngeal carcinoma. Eligible patients were randomized 1:1 to the control group and the edaravone group (intravenous 30 mg twice per day for 2 weeks). Both groups received intravenous conventional steroid therapy and were monitored by brain MRI and LENT/SOMA scales prior to the entry of the trial and at 3-months after completing the trial. The primary end point was a 3-month response rate of the proportional changes determined by MRI. The trial is registered at Clinicaltrials.gov Identifier: NCT01865201. Between 2009 and 2012, we enrolled 154 patients. Of whom 137 were eligible for analysis. The volumes of necrosis estimated on T_2_-weighted image showed that 55.6 % edaravone-treated patients (40 out of 72) showed edema decreases ≥25 %, which was significantly higher than that in the control group (35.4 %, 23 out of 65, *p* = 0.025). Forty-four patients treated with edaravone (61.1 %) reported improvement in neurologic symptoms and signs evaluated by LENT/SOMA scales, while the rate was 38.5 % in the control group (*p* = 0.006). MRI of the edaravone group showed a significant decrease in area of T_1_-weighted contrast enhancement (1.67 ± 4.69 cm^2^, *p* = 0.004) and the T_2_-weighted edema (5.08 ± 10.32 cm^2^, *p* = 0.000). Moreover, compared with those in control group, patients with edaravone exhibited significantly better radiological improvement measured by T_2_-weighted image (*p* = 0.042). Administration of edaravone, in adjunct to steroid regimen, might provide a better outcome in patients with radiation-induced brain necrosis.

## Introduction

Radiation-induced brain necrosis (RN) is a serious complication caused by radiation therapy. Occurrence of RN can be acute, sub-acute, and delayed, which adversely worsens neurologic functions and impairs the quality of life of recipients. Corticosteroids are commonly used as a protective agent for RN. However they have significant side effects depending on the dose and duration of exposure. Side effects can include diabetes, iatrogenic Cushing syndrome, myopathy, avascular necrosis, psycho-mood disturbance, etc. [1]. Although corticosteroids may ameliorate the severity of the signs and symptoms related to RN, the effect may lost once the steroids are discontinued [2]. Alternative therapies such as hyperbaric oxygen therapy, antiplatelet agents, anticoagulants, and/or vitamins have clinically been tested but the outcomes are disappointing. Antiangiogenic agent, bevacizumab has recently been reported to be promising [3, 4], yet with flaw of potential toxicity and high cost. Surgical debulk is one of the treatment options but many RN lesions are inoperable because of the location, or the fact that patients may be medically illegible for surgery [5–7].

The pathogenesis of RN is under exploration but not fully understood. It has been confirmed that chronic oxidative stress and inflammatory reaction play key roles in the pathogenesis of radiation-induced late normal tissue injury [8, 9]. Free radicals can be generated directly or indirectly by ionizing radiation [10, 11]. Ample evidence from laboratory experiments revealed that excessive generation of free radicals causes tissue damage in many ways by which they react on protein, lipid, and double DNA strands resulting in metabolic disturbances and cellular death. Specifically, the central nervous system (CNS) is vulnerable to oxidative stress, therefore, eliminating the insulting effects induced by free radicals, may be beneficial in alleviation of cellular damage.

Edaravone (3-methyl-1-phenyl-2-pyrazolin-5-one), as an effective free radical scavenger [12–15], has been used in treating a wide range of oxidative stress-related diseases, including cerebral ischemia, by scavenging free radicals and suppressing the inflammatory reactions [16]. However, whether edaravone has a therapeutic effect on RN is unknown. To test this hypothesis, we carried out a prospective randomized open-labeled clinical study on the effect of edaravone on RN.

## Methods

This prospective study was a 3-month, open-labeled clinical trial on RN comparing the patients treated with edaravone plus corticosteroid to control group treated with corticosteroid alone. The study was performed at the Department of Neurology of Sun Yat-Sen Memorial hospital, Sun Yat-Sen University, and was approved by the authorized human research review board in our institutes in accordance with the Helsinki declaration. Written informed consents were obtained from all participants.

### Participants

Patients were enrolled between March 2009 and September 2012. The inclusion criteria were as follows: ①Have received radiotherapy for histologically confirmed nasopharyngeal carcinoma; ②Radiotherapy was finished ≥6 months prior to study entry; ③Radiographic evidence to support the diagnosis of RN without tumor recurrence [17]; ④Age ≥ 18 years; ⑤No evidence of increased intracranial pressure suggestive of a brain herniation; ⑥Routine laboratory studies with bilirubin ≤2*upper limits of normal (ULN), aspartate aminotransferase (AST or SGOT) <2* ULN, creatinine <1.5*ULN, red-cell count ≥4,000 per cubic millimeter; white-cell count ≥1500 per cubic millimeter, platelets ≥75,000 per cubic millimeter; Hb ≥ 90 g/L, prothrombin time(PT), activated partial thromboplastin time(APTT), international normalized ratio(INR) in a normal range; ⑦Being able to understand and willing to sign a written informed consent document. Considering the potential side effects of edaravone on liver function, after the trial was started, we decided to enroll patients with normal AST. The exclusion criteria were as follows: ①Evidence of tumor recurrence or metastases; ②Other CNS disorders, such as cerebral vascular events, inflammatory and neurodegenerative diseases; ③Concomitant significant systemic diseases such as cardiovascular diseases; ④History of anaphylactic response to edaravone. All participants have received radical radiotherapy using a conventional two-dimensional radiotherapy technique.

### Interventions

Participants were randomized in the ratio of 1:1 into the control group and the edaravone group. All participants received a conventional corticosteroid regimen (administration of daily intravenous methylprednisolone 500 mg for 3 consecutive days followed by oral prednisolone tapering down to off in 30 days. Edaravone was given at 30 mg, twice per day for 14 days as a reference of the dosage used in patients with ischemic stroke [18]. Demographic data, total dose (Gy), diagnosis, treatments, clinical and radiographic responses of participants were collected.

### Outcomes

Clinical symptoms and signs were evaluated by Late Effects of Normal Tissues –Subjective, Objective, Management, Analytic (LENT/SOMA) scale [19] before entry of the trial and 3 months after treatment. Subjective domain contains five items: headache, somnolence, intellectual deficit, functional competence, and memory. Objective domain contains four items: neurologic deficit, cognitive functions, mood & personality changes, and seizures. Analytic domain includes neuropsychologic and radiological assessments. Each parameter scores from 1 to 4, score 0 if there are no toxicities. The total of each parameter represents the final score of the LENT/SOMA scale [19]. The radiological response was assessed by MRI difference between pretreatment and post-treatment, which included T_1_-weighted gadolinium contrast-enhanced and T_2_-weighted image. The edge of the maximum area of each lesion was draw by the manual approach and calculated automatically by software Volume Viewer 2(GE, AW Suite 2.0, 6.5.1.z). MRI studies were evaluated by two neuroradiologists who were blinded to the grouping.

The primary endpoint was the proportion of the edema reduction in area at 3 months. A reduction in edema area of ≥25 % constituted a response, which was estimated on T_2_-weighted images. The LENT/SOMA scale score was selected as the secondary endpoint measures.

### Statistical analysis

We designed the present study with an α of 0.05, and power of 90 %. In order to detect an increase of 30 % in response rate between the two arms, the estimated sample size for this study was 112 patients (56 patients in each arm). The data were expressed as mean ± standard deviation (SD). T test was used to compare age, radiation dose, latency of RN between the edaravone group and the control group. LENT/SOMA scale scores between the two groups were assessed by Mann–Whitney* U* Test. The χ^2^ test was used to analyze the therapeutic efficacy. Differences in the T_1_-weighted gadolinium enhancement and T_2_-weighted image between the two groups were also evaluated by *t* test. A *p* value of less than 0.05 was accepted as significant.

## Results

### Demographic data

**Fig. 1:**
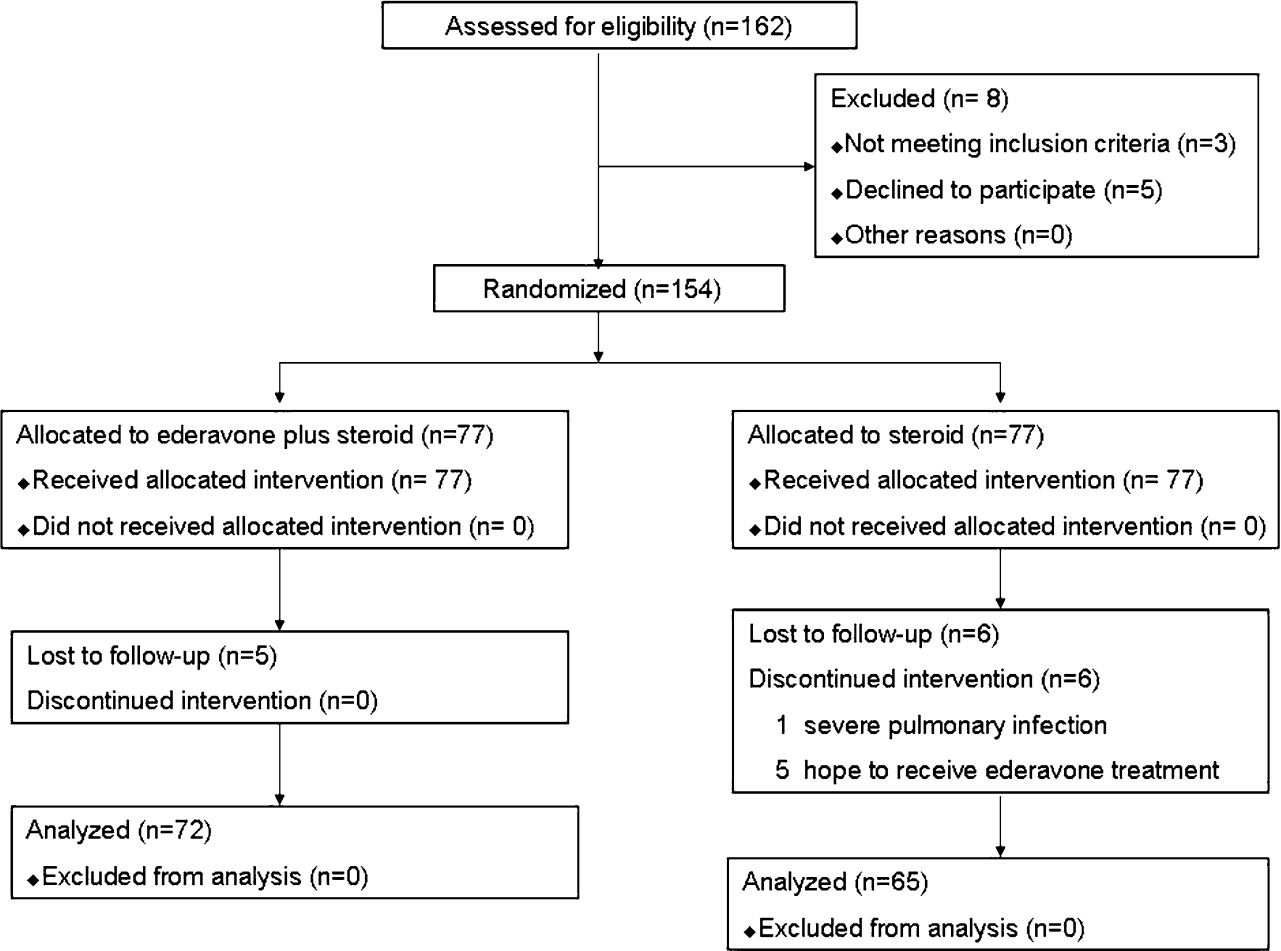
Flow diagram of our study

Between March 2009 and September 2012, a total of 162 patients were added to the study. Of these, 154 who fulfilled the inclusion criteria were enrolled in the study. Seventy-seven were enrolled in the edaravone-treated group and another 77 in the control group. During the study, 17 (5 in edeverone group and 12 in control group) withdrew. In control group, one patient suffered from severe pulmonary infection and could not continue the corticosteroid therapy. Five patents in the control group asked for edaravone, therefore also withdrew from the protocol. A total of 11 patients were lost to follow-up. The final number of the subjects in the study was 137, namely 72 in edavarone group and 65 in control group, as shown in Fig. 1.

All participants received radiotherapy at a total dose of 68–82 Gy (2 Gy/fraction, 5 fractions per week). The patients’ age ranged from 25 to 80 years old, with an average of 50.6 ± 10 years. The latency from radiotherapy to diagnosis of RN was 5.4 ± 4.2 years. The most frequent symptoms were headache, dizziness, fatigue, dysphasia, hearing loss, memory decline, personality change, and seizures. Twenty-seven patients (19.7 %) had clinical evidence of cranial nerve palsy. Clinical data of the two groups are summarized in Table 1. There were no statistical differences between the two groups regarding age, gender, latency of RN, total radiation dose, scores of LENT/SOMA scale at baseline, and maximum area of necrosis lesions.

**Table 1:** Baseline characteristics of the study population

	Edaravone group (*n* = 72)	Control group (*n* = 65)	*p* value
Age (year)	49.2 ± 9.2	52.0 ± 11.0	0.099
Gender			
Male	56	52	0.835
Female	16	13	
Primary tumor	NPC	NPC	1.000
Cranial nerve palsy	14	13	1.000
Latency of RN (year)	5.6 ± 4.6	5.1 ± 3.7	0.508
Conventional RT (Gy)	72.6 ± 3.1	71.4 ± 3.3	0.052
LENT/SOMA scores			
6	54	51	0.558
7	14	13	
8	4	1	
T_1_-weight gadolinium-contrast area (cm^2^)	6.77 ± 5.09	7.68 ± 4.77	0.286
T_2_-weithted area (cm^2^)	19.42 ± 10.54	22.58 ± 10.09	0.075

*NPC* nasopharyngeal carcinoma, *RN* radiation-induced brain necrosis, *RT* radiotherapy

### MRI observations

**Fig. 2:**
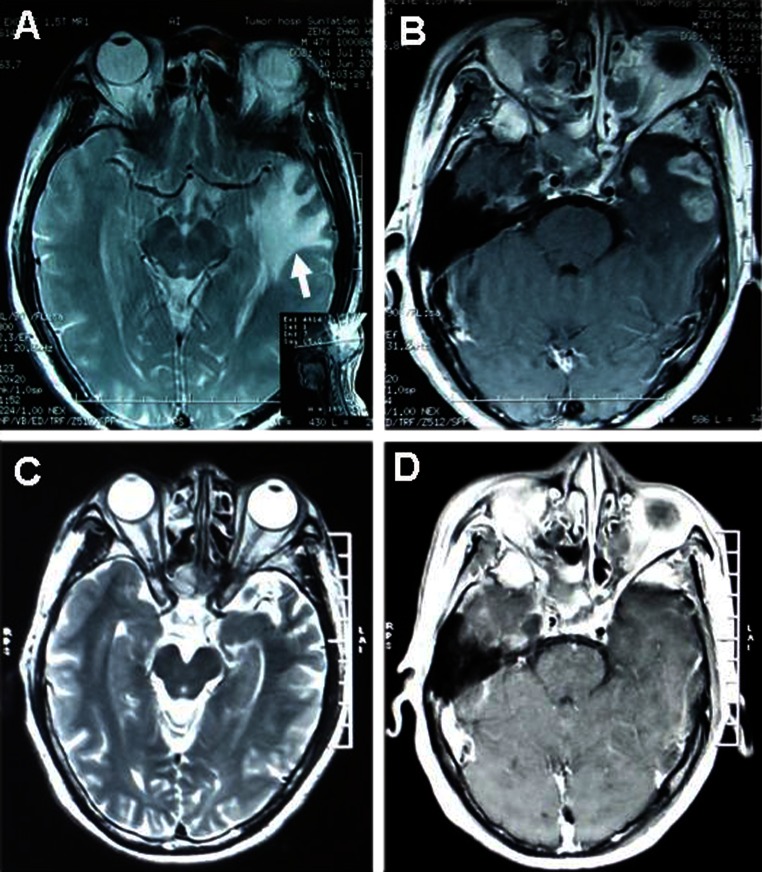
MRI of one patient in edaravone group. **a**, **b** showed *left* temporal lobe necrosis (*arrow head*) before treatment. Three months after treatment, T_2_-weighted edema (**c**) and T_1_-weighted gadolinium contrast-enhancement (**d**) were significantly reduced

MRI scan was obtained at the baseline prior to the entry of the trial and 3 months after completing the trial. Forty patients in edaravone group and twenty-three patients in control group exhibited radiological improvement (55.6 vs. 35.4 %, *p* = 0.025). Though both two groups showed significantly decreased edema area measured in T_2_-weighted MRI (edaravone group 5.08 ± 10.32 cm^2^, *p* = 0.000; control group 2.05 ± 6.71 cm^2^, *p* = 0.016), more reduced edema area was demonstrated in edaravone group compared with that in the control group (*p* = 0.042). Notably, patients in both groups showed a significant decrease in the size of the T_1_-weighted gadolinium enhancement (edaravone group, 1.67 ± 4.69 cm^2^, *p* = 0.004; control group, 1.20 ± 2.71 cm^2^, *p* = 0.001, Table 2), but it did not reach the statistical difference between the two groups (*p* = 0.468, Table 2). Figure 2 showed a representative images obtained in a patient with edaravone.

**Table 2:** Efficacy of Improvement Evaluated by LENT/SOMA scale and MRI

	Edaravone group (*n* = 72) (%)	Control group (*n* = 65) (%)	*p* value
LENT/SOMA			
Improved	44 (61.1)	25 (38.5)	0.006
Ineffective	28 (38.9)	40 (61.5)	
MRI			
Reduction in T_1_ + C (cm^2^)	1.67 ± 4.69	1.20 ± 2.71	0.468
Reduction in T_2_ (cm^2^)	5.08 ± 10.32	2.05 ± 6.71	0.042

*T*_*1*_ *+* *C* T_1_-weighted gadolinium contrast enhancement

### LENT/SOMA scale results

LENT/SOMA scale was evaluated before treatment and 3 months after treatment. A reduction in score of the LENT/SOMA scale of ≥1 constituted an improvement. Forty-four in the edaravone group and 25 control group patients showed clinical improvement, from which a significantly better response was observed in edaravone group than that of the control group (61.1 vs. 38.5 %, *p* = 0.006, Table 2).

### Adverse events

The adverse events are listed in Table 3. Nine of 72 patients in edaravone group and 10 of 65 in the control group had adverse events, without significant difference between two groups. Of these 19 events, none of them were serious: seven were insomnia, six high blood glucose, three epistaxis, two mild dysfunction of liver and one hypertension.

**Table 3:** Adverse events in the two groups

	Edaravone group (*n* = 72) (%)	Control group (*n* = 65) (%)	*p* value
Epistaxis	1 (1.4)	2 (3.1)	0.604
High blood glucose	3 (4.2)	3 (4.6)	1.000
Dysfunction of liver*	2 (2.7)	0 (0.0)	0.498
Hypertension	0 (0.0)	1 (1.5)	0.474
Insomnia	3 (4.2)	4 (6.2)	0.708

* Dysfunction of liver was defined as aspartate aminotransferase or alanine aminotranferase >/= 2 * upper limits of normal

## Discussion

RN is a serious complication of radiotherapy. It may cause significant focal dysfunction and/or cognitive impairment. The incidence of RN was reported to be 4.6 % in 10 years to 35 % in 3.5 years after radiotherapy [20]. Although corticosteroid is employed as the major conventional therapeutic agent for RN, its efficacy remains unsatisfactory [21].

Previous studies have shown that the free radical scavenger, edaravone, bears neuroprotective effect in some oxidative stress-related diseases [16, 22, 23]. The amenable mechanism of edaravone in this regard is believed to suppress the production of free radicals and scavenge reactive oxidative species in the brain [24]. This anti-oxidative property protects cellular structures from oxidative injury and cell death because oxidative stress has been confirmed in mediating radiation-induced injury [25]. It may also prevent the neural precursor cells from apoptosis in the neurogenesis zone of the brain [26, 27]. In addition, edaravone inhibits the expression of vascular endothelium growth factor in astrocytes [28], and has been shown to protect neurons from cell death after irradiation [27]. These observations suggest that edaravone may have a protective effect on the brain from development of RN.

In our study, patients in both edaravone and control groups exhibited a significant reduction in T_1_-weighted contrast enhancement and T_2_-weighted edema. Moreover, edaravone-treated patients have a significantly reduced edema area compared with that of patients from control group, indicating that adding edaravone to corticosteroid regimen can enhance radiological improvement relevant to RN. Although no statistical significance was observed in T_1_-weighted image between the two groups in our study, it may be due to the small size of the samples. Our results also demonstrated that clinical symptoms did not always parallel with the MR imaging. Some patients might have symptoms virtually gone without significant recovery of necrosis lesions.

Several therapeutic agents adjunct to the conventional corticosteroid regimen have been tested for RN in the past two decades (e.g. anticoagulants, anti-platelets, hyperbaric oxygen therapy), but their use is not widely accepted because of the potential adverse effects and limited randomized control trials. Since radiotherapy on head and neck may cause carotid stenosis [29], intracranial hemorrhage or epistaxia, the therapeutic agent adjunct to corticosteroid must be safe from leading to ischemic or hemorrhagic events. Edaravone has been reported to be effective in ischemic stroke and aneurismal subarachnoid hemorrhage [23], it may be safe in RN too. In our study, the fact that no fatal or severe adverse events were observed in the edaravone group supported our notion.

In conclusion, our results suggested that administration of edaravone adjunct to the conventional corticosteroid regimen may be beneficial in reduction of RN. To validate the neuroprotective effect of edaravone, a large scale study is warranted, and the long term effect of edaravone therapy should be elucidated. In addition, the edaravone-treated patients needed additional charges for the drug, although they had a better clinical outcome. Thus, cost-benefit analysis is also recommended for its further usage.

## References

[1] Lam TC, Wong FC, Leung TW, Ng SH, Tung SY (2012). Clinical outcomes of 174 nasopharyngeal carcinoma patients with radiation-induced temporal lobe necrosis. Int J Radiat Oncol Biol Phys.

[2] Chen J, Dassarath M, Yin Z, Liu H, Yang K (2011). Radiation induced temporal lobe necrosis in patients with nasopharyngeal carcinoma: a review of new avenues in its management. Radiat Oncol.

[3] Wong ET, Huberman M, Lu XQ, Mahadevan A (2008). Bevacizumab reverses cerebral radiation necrosis. J Clin Oncol.

[4] Levin VA, Bidaut L, Hou P, Kumar AJ, Wefel JS (2011). Randomized double-blind placebo-controlled trial of bevacizumab therapy for radiation necrosis of the central nervous system. Int J Radiat Oncol Biol Phys.

[5] Glantz MJ, Burger PC, Friedman AH, Radtke RA, Massey EW (1994). Treatment of radiation-induced nervous system injury with heparin and warfarin. Neurology.

[6] Hampson NB, Holm JR, Wreford-Brown CE, Feldmeier J (2012). Prospective assessment of outcomes in 411 patients treated with hyperbaric oxygen for chronic radiation tissue injury. Cancer.

[7] Mou YG, Sai K, Wang ZN, Zhang XH, Lu YC (2011). Surgical management of radiation-induced temporal lobe necrosis in patients with nasopharyngeal carcinoma: report of 14 cases. Head Neck.

[8] Kim JH, Brown SL, Jenrow KA, Ryu S (2008). Mechanisms of radiation-induced brain toxicity and implications for future clinical trials. J Neurooncol.

[9] Robbins ME, Zhao W (2004). Chronic oxidative stress and radiation-induced late normal tissue injury: a review. Int J Radiat Biol.

[10] Dizdaroglu M, Jaruga P, Birincioglu M, Rodriguez H (2002). Free radical-induced damage to DNA: mechanisms and measurement. Free Radic Biol Med.

[11] Wallace SS (2002). Biological consequences of free radical-damaged DNA bases. Free Radic Biol Med.

[12] Perez-Gonzalez A, Galano A (2011). OH radical scavenging activity of Edaravone: mechanism and kinetics. J Phys Chem B.

[13] Perez-Gonzalez A, Galano A (2012). On the outstanding antioxidant capacity of edaravone derivatives through single electron transfer reactions. J Phys Chem B.

[14] Ahmad A, Khan MM, Javed H, Raza SS, Ishrat T (2012). Edaravone ameliorates oxidative stress associated cholinergic dysfunction and limits apoptotic response following focal cerebral ischemia in rat. Mol Cell Biochem.

[15] Borges RS, Queiroz AN, Mendes AP, Araujo SC, Franca LC (2012). Density functional theory (DFT) study of edaravone derivatives as antioxidants. Int J Mol Sci.

[16] Watanabe T, Tahara M, Todo S (2008). The novel antioxidant edaravone: from bench to bedside. Cardiovasc Ther.

[17] Chen J, Dassarath M, Yin Z, Liu H, Yang K (2011). Radiation induced temporal lobe necrosis in patients with nasopharyngeal carcinoma: a review of new avenues in its management. Radiat Oncol.

[18] Nakase T, Yoshioka S, Suzuki A (2011). Free radical scavenger, edaravone, reduces the lesion size of lacunar infarction in human brain ischemic stroke. BMC Neurol.

[19] Fehlauer F (1995). LENT SOMA scales for all anatomic sites. Int J Radiat Oncol Biol Phys.

[20] Chen J, Dassarath M, Yin Z, Liu H, Yang K (2011). Radiation induced temporal lobe necrosis in patients with nasopharyngeal carcinoma: a review of new avenues in its management. Radiat Oncol.

[21] Lam TC, Wong FC, Leung TW, Ng SH, Tung SY (2012). Clinical outcomes of 174 nasopharyngeal carcinoma patients with radiation-induced temporal lobe necrosis. Int J Radiat Oncol Biol Phys.

[22] Yoshino H, Kimura A (2006). Investigation of the therapeutic effects of edaravone, a free radical scavenger, on amyotrophic lateral sclerosis (Phase II study). Amyotroph Lateral Scler.

[23] Munakata A, Ohkuma H, Nakano T, Shimamura N, Asano K (2009). Effect of a free radical scavenger, edaravone, in the treatment of patients with aneurysmal subarachnoid hemorrhage. Neurosurgery.

[24] Firuzi O, Miri R, Tavakkoli M, Saso L (2011). Antioxidant therapy: current status and future prospects. Curr Med Chem.

[25] Zhang Y, Zhang X, Rabbani ZN, Jackson IL, Vujaskovic Z (2012). Oxidative stress mediates radiation lung injury by inducing apoptosis. Int J Radiat Oncol Biol Phys.

[26] Ishii J, Natsume A, Wakabayashi T, Takeuchi H, Hasegawa H (2007). The free-radical scavenger edaravone restores the differentiation of human neural precursor cells after radiation-induced oxidative stress. Neurosci Lett.

[27] Motomura K, Ogura M, Natsume A, Yokoyama H, Wakabayashi T (2010). A free-radical scavenger protects the neural progenitor cells in the dentate subgranular zone of the hippocampus from cell death after X-irradiation. Neurosci Lett.

[28] Ishikawa A, Yoshida H, Metoki N, Toki T, Imaizumi T (2007). Edaravone inhibits the expression of vascular endothelial growth factor in human astrocytes exposed to hypoxia. Neurosci Res.

[29] Marcel M, Leys D, Mounier-Vehier F, Bertheloot D, Lartigau E (2005). Clinical outcome in patients with high-grade internal carotid artery stenosis after irradiation. Neurology.

